# Growth of CNT Forests on Titanium Based Layers, Detailed Study of Catalysts

**DOI:** 10.3389/fchem.2018.00593

**Published:** 2018-12-03

**Authors:** Anna Szabó, Pavao Andricević, Zsuzsanna Pápa, Tamás Gyulavári, Krisztián Németh, Endre Horvath, László Forró, Klara Hernadi

**Affiliations:** ^1^Department of Applied and Environmental Chemistry, University of Szeged, Szeged, Hungary; ^2^Laboratory of Physics of Complex Matter (LPMC), Ecole Polytechnique Federale de Lausanne, Lausanne, Switzerland; ^3^Department of Optics and Quantum Electronics, University of Szeged, Szeged, Hungary

**Keywords:** CNT forests, conductive substrate, CCVD synthesis, titanium substrate, Fe-Co catalyst, hybrid perovskite photodetectors

## Abstract

For better electrical contacts of potential devices, growth of vertically aligned carbon nanotubes (CNT forests) directly onto conductive substrates is an emerging challenge. Here, we report a systematic study on the CCVD synthesis of carbon nanotube forests on titanium based substrates. As a crucial issue, the effect of the presence of an insulating layer (alumina) on the growing forest was investigated. Other important parameters, such as the influence of water vapor or the Fe-Co catalyst ratio, were also studied during the synthesis. As-prepared CNT forests were characterized by various techniques: scanning and transmission electron microscopies, Raman spectroscopy, spectroscopic ellipsometry. CNT forests grown directly onto the conductive substrate were also tested as electrodes in hybrid halide perovskite photodetectors and found to be effective in detecting light of intensity as low as 3 nW.

## 1. Introduction

Vertically aligned carbon nanotubes (VACNT) which are often referred to as carbon nanotube forests in the literature (CNT forest), were synthesized for the first time in 1996 (Li et al., 1996). Since then, this invention has served as a novel architectural design to integrate into various devices in the field of nanotechnology. The most conventional way to produce CNT forests is *via* catalytic chemical vapor deposition (CCVD). During the CVD method, the most commonly used catalysts are transition metals, which can be Fe, Co, Ni, while SiO_2_, Al_2_O_3_, or MgO are often used as oxide support (Noda et al., 2007; Halonen et al., 2008; Mattevi et al., 2008; Sakurai et al., 2011; Robertson et al., 2012). In the research field of CNT forests an important breakthrough was made in 2004, when Hata et al. (2004) introduced a small amount of water into the CVD synthesis chamber, which drastically influenced the growth rate the ultimate height and quality of VACNT. Although, there is a growing understanding about the molecular-level mechanism of this so called “super-growth” method, still studying the influence of the synthesis conditions on the physicochemical properties of CNTs is still crucial, in order to reveal and tune the parameter space of the properties such as the orientation, the height, the density, and degree of graphitization.

The catalyst layer can be deposited in various ways. For example, wet-chemical methods as dip-coating, spray coating, or high vacuum techniques as thermal evaporation, magnetron sputtering, and pulsed laser deposition (PLD) (Mauron et al., 2002; Murakami et al., 2003; Fejes et al., 2015). In 2007, Noda et al. studied the effect of the presence of aluminum oxide on silicon substrate in relation to the synthesis of carbon nanotube forests. They have found that the intermediate oxide support on the silicon substrate was crucial to provide a strong interaction between the oxide layer and the catalyst layer.

Besides silicon, many other materials, such as SiO_2_, stainless steel, copper, aluminum, and titanium could serve as a support for VACNT synthesis (Santhanagopalan et al., 2009; Atthipalli et al., 2011; Dörfler et al., 2013; Zhu et al., 2013; Silva et al., 2014). Potential use of VACNTs in electronics and optoelectronics aims for the elimination of the insulating oxide layer and necessitates the growth of carbon nanotubes directly on conductive substrates to provide better electrical contact. Only few publications are addressing this topic, where the synthesis of the carbon nanotube forests was achieved directly on metallic aluminum or stainless steel. The aim of these papers was mainly to investigate the conductivity properties of the products (Matthews et al., 2006; Masarapu and Wei, 2007; Pattinson et al., 2015).

Regarding the synthesis of carbon nanotube forests, the formation of the catalyst layer is a significant parameter, hence it strongly affects the growth of the carbon nanotubes. Several publications have dealt with the effect of catalyst ratios, the most commonly used transitional metals were such as Fe, Co, and Ni (Dresselhaus et al., 2005; Antunes et al., 2006). In the literature, Fe:Co = 1:1 is most often used (Shokry et al., 2014), nevertheless FeO also has contributed to the growth of carbon nanotube forests, where iron oxide clusters were formed on the substrate (Mauron et al., 2002). However, thorough research was carried out in this topic, where other ratios have been studied and such an observation could be made that similar results can be achieved using other catalyst ratios (Seo et al., 2003; Magrez et al., 2011; Szabó et al., 2017).

Here, we investigate the effect of aluminum oxide support on the growth of carbon nanotube forests over metallic titanium substrates. The as prepared VACNTs have been combined with organic inorganic lead halide perovskite single crystals to prepare heterojunction interfaces by applying simple mechanical pressure and point-contact electrodes. We have found that the VACNTs without alumina layer could serve as an excellent electrode material for lead halide perovskite photodetectors. The elimination of the alumina deposition step might reduce the degree of complications, ultimately the price in the photodetector fabrication process.

## 2. Materials and methods

### 2.1. Materials

In the experimental part Titanium sheets were used, manufactured by WRS Materials Company. The catalyst layers were evolved using aluminum-oxide (WRS Materials Company), iron (III)-oxide (99.998%, Sigma-Aldrich), and cobalt (II)-oxide (99.99%, Sigma-Aldrich) were used as pellets. During CCVD synthesis ethylene, hydrogen, and nitrogen were used, all manufactured by Messer Hungary.

### 2.2. Catalyst layer production

Catalyst layers and oxide were prepared by PLD following the same deposition conditions as in our previous work (Pápa et al., 2018). Catalyst and oxide target were made of metal oxides' powder (Fe_2_O_3_, CoO and Al_2_O_3_) with a total weight of 1 g shaped into a 1 cm diameter pellet. The mechanical resistance was improved by heat treatment, which in this case lasted 4 h at 500°C in air. In order to provide reproducible adhering conditions onto the substrate, the titanium substrate was sequentially washed with distilled water, absolute ethanol, and acetone prior to catalyst layer deposition. The cleaned titanium substrate was placed into a vacuum chamber. For the layer deposition, laser pulses of a LLG TWINAMP ArF excimer laser (λ = 193 nm, pulse length: 18 ns, repetition rate: 10 Hz) with average fluence of 13 J/cm^2^ were focused on the target pellets placed in front of the titanium substrate where the layer was formed. The target-substrate distance was 3 cm. The catalyst layer thickness could be tuned with the number of lasers shots. According to a previous thickness optimization, the catalyst layer thickness was set to be 5 nm proven by spectroscopic ellipsometry measurements (Woollam M-2000F) (Fejes et al., 2015; Pápa et al., 2018).

### 2.3. CCVD synthesis

For the carbon nanotube forest production, the CCVD synthesis method was used. The titanium sheets including the catalyst layers were cut into 4 × 4 mm small sheets, in order to fit the quartz boat, (diameter 20 mm). The experiments were carried out at 700°C and the reaction time was 30 min. During the synthesis, the carrier gas was nitrogen with a flow rate of 50 cm^3^/min, the carbon source was ethylene with a flow rate of 70 cm^3^/min, the reducing agent was hydrogen with a flow rate of 50 cm^3^/min, while the system contained water vapor with a flow rate of 30 cm^3^/min, which contributed to the growth of carbon nanotube forests.

In the first step of the synthesis, the reactor was purged with nitrogen to exclude oxygen from the system (2 min). Then hydrogen gas was introduced into the reactor, to reduce the catalysts (5 min). Subsequently, ethylene and water vapor were added to the synthesis. When the reaction was finished, all gas flows were closed, except nitrogen gas, which remained in the system for an additional 5 min. After the reactor was removed from the oven, and it was cooled to room temperature; in the final step as-synthesized samples were removed from the reactor. “Blank” synthesis was also carried out with the elimination of carbon source ethylene.

### 2.4. Microscopic and spectroscopic characterization of CNT samples

The orientation of the CNT forests was investigated by the means of Scanning Electron Microscopy (SEM), which type was Hitachi S-4700 Type II FE-SEM (5–15 keV). For the careful measurement of CNTs, the sample holder was tilted at a 35° angle within the SEM device, making possible their examination from all directions. The SEM results were evaluated with ImageJ software. During determination of the height of CNT forests this condition has to be taken into account, thus the measured height was divided by sin 35° based on geometric considerations to obtain the actual height.

The diameters of the carbon nanotubes were examined by Transmission Electron Microscopy (TEM, Philips CM 10, 100 keV). In order to prepare the TEM grids, small amount of CNT forests were scraped off the Ti foil with a spatula and was suspended in 1.25 cm^3^ absolute ethanol. Two to three drops of the suspension were applied on the holey carbon grid (Lacey, CF 200).

The graphitic properties of CNT were analyzed by Raman Spectroscopy (Thermo Scientific DXR Raman microscope, excitation wavelength 532 nm).

### 2.5. Fabrication of hybrid perovskite photodetectors

#### 2.5.1. Crystal growth

Crystals of the methylammonium lead tribromide were synthesized by solution growth. The 3.3 mmol lead (II) acetate trihydrate (Pb(ac)_2_ × 3*H*_2_*O*, > 99.9%) was reacted with 6 ml saturated HBr solution (48 wt% HBr in H_2_O). The formed PbBr_2_ precipitate is stable in the acidic solution. The respective amount (3.30 mmol) of methylamine (CH_3_NH_2_) solution (40 wt% in H_2_O) was pipetted into the 5 °C ice cooled solution of PbBr_2_. The cold solution avoids the evaporation of methylamine during the exothermic reaction. Orange colored microcrystallites of CH_3_NH_3_PbBr_3_ were formed. The MAPbBr_3_ crystals were recrystallized in a temperature gradient of 15°C in the acidic media to get transparent, high purity crystals.

#### 2.5.2. Optoelectronic characterization

All the performances measurements of the devices were done in ambient conditions at room temperature. The junction characteristics have been determined by two points resistivity measurements, tungsten needles as electrical leads. One of the contacts is positioned directly on the perovskite single crystal, whereas the second one touches the Ti foil as the back electrode. A Keithley 2400 source meter allowed us to measure the current with < 0.1 nA resolution, while tuning the applied bias voltage, in dark and under visible light illumination. Current-Voltage measurements were performed by sweeping the voltage from 0 to +2 V/-2 V and back, with a scan speed of 0.2 V/s. Photocurrent measurements at low light intensities were done by choosing 550 nm wavelength, within the spectral response of our device, enabling also to achieve high enough intensities of light that can be detected. The wavelength was set with a monochromator (Horiba Micro HR), while light intensity was adjusted by closing and opening slits in the light path.

## 3. Results

### 3.1. The effect of the oxide layers on the titanium-based support

Firstly, the presence of the oxide layer on the surface of the substrate was investigated regarding its influence on the quality of the CNT forests. During the synthesis two possibilities were inspected: applying an Al_2_O_3_ layer between the catalyst layer and the substrate, and in the other case, without any oxide layer present. All synthesis parameters were kept identical, including the thickness of the catalyst layer of 5 nm.

**Figure 1:**
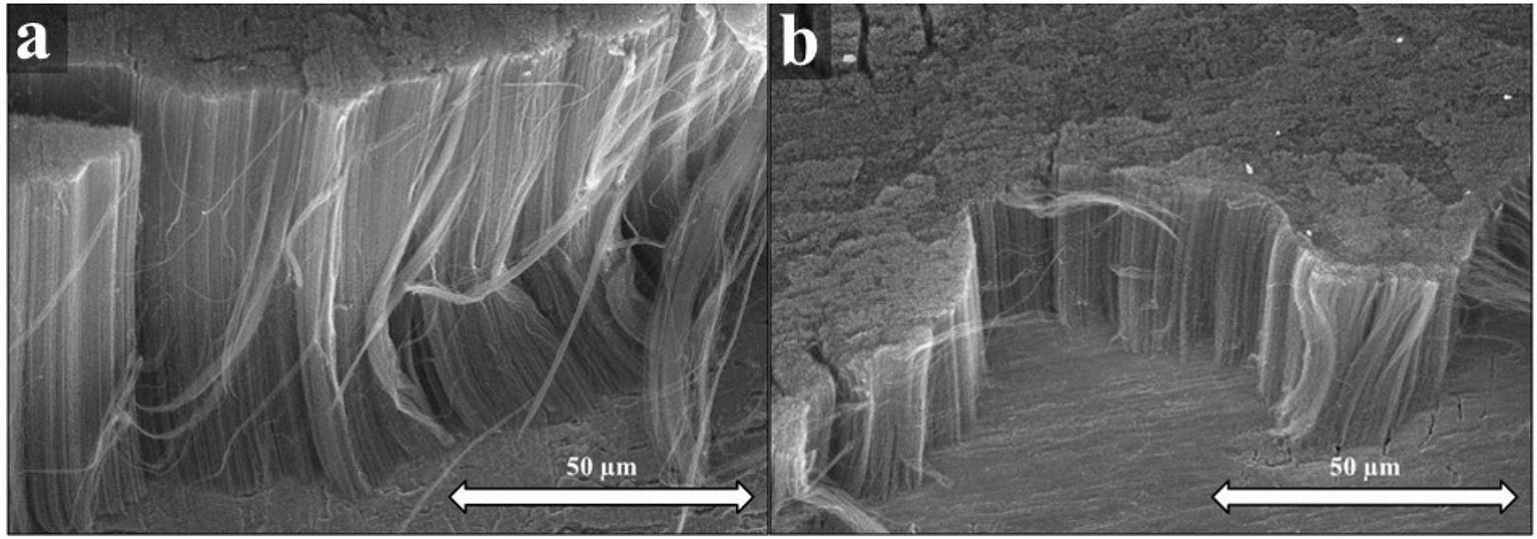
SEM images of CNT forests synthesized with Al_2_O_3_ oxide support **(a)**, SEM images of CNT forests synthesized without Al_2_O_3_ oxide support **(b)**.

In literature, oxide layers are often used on the substrate, as they may influence the CNT forests quality significantly *via* promoting the separation of catalytic particles. The interface created between the Al_2_O_3_ and the catalyst particles was proved to play an essential role on the growth of CNTs. Furthermore, the hydrocarbon adsorption onto the aluminum oxide, and the surface diffusion from the aluminum oxide to the Fe nanoparticles was found to be very important (Noda et al., 2007). Nevertheless, CNT forests grown directly on metals might allow immediate junction with the conductive substrate, which can result in reduced contact resistance providing increased conductivity of the sample (Zhao and Kang, 2011). The effect of oxide supports on the CNT forest was first characterized by SEM images (Figure 1).

Neverthless, the CNT forests can clearly grow on a conductive support without an aluminum oxide layer, as seen in the SEM image (Figure 1b). However, it could be observed that the height of CNT forests was influenced significantly by the oxide support. While the height of the CNT forests over the alumina support was 108 μm (Figure 1a), the height of the CNT forests over metallic titanium support was only 32 μm (Figure 1b). However, besides the disparity in height no considerable difference in the quality was detected according to the SEM micrographs. However, water vapor might oxidize the metallic substrates *in-situ* during the nanotube growth.

Therefore, to exclude potential artifacts, the possibility of growing carbon nanotube vertically aligned structures on metallic titanium substrate without water vapor was tested. Samples with and without the intermediate alumina layer were prepared. The synthesis conditions were kept constant, as before, with the addition of absolutely excluding water vapor from the feed to prove whether the oxidative property of water vapor was indispensable during the synthesis.

**Figure 2:**
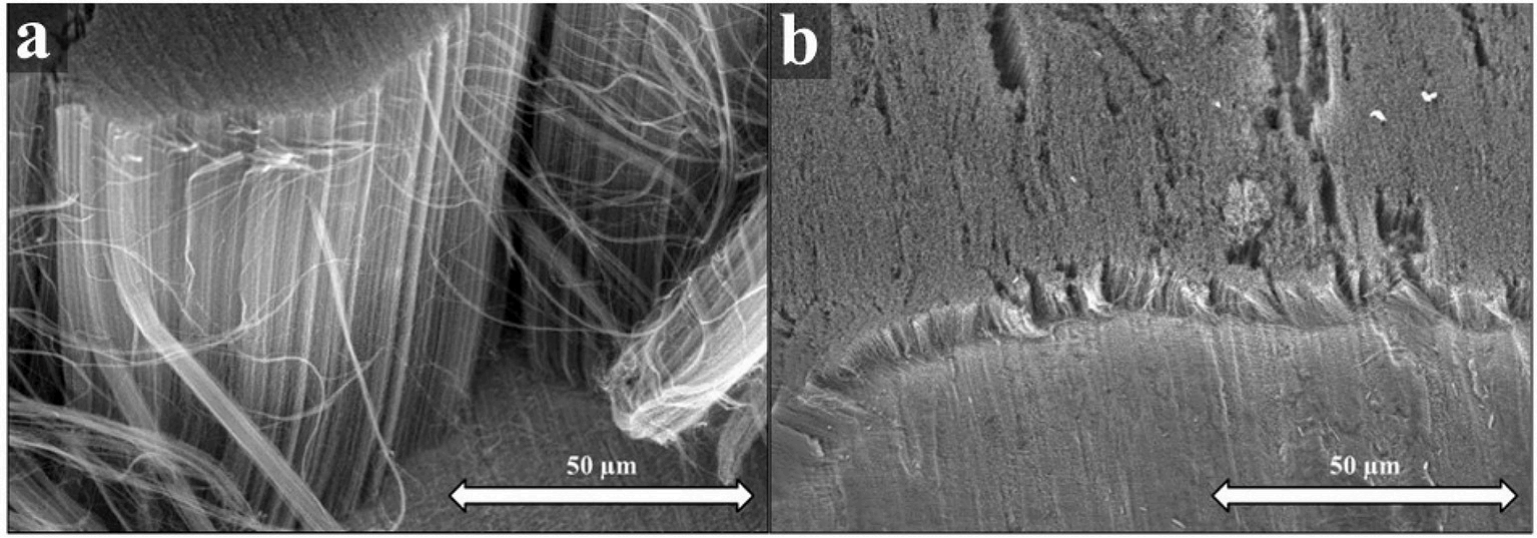
SEM images of CNT forests synthesized with **(a)** and without **(b)** Al_2_O_3_ oxide support with no water vapor in the gas feed.

Again, it is clearly visible from SEM images that carbon nanotube forests are formed on both types of Ti substrates (Figure 2) Interestingly, nevertheless the height of the forest on the titanium substrate having the alumina intermediate layer is in the same range (Figure 2) as that one synthesized with water vapor. Unlike the catalyst having the alumina layer, those without alumina showed significantly reduced height as compared their counterparts prepared in the presence of water vapor. While the height of carbon nanotube forest was 32 μm in the presence water vapor, this value dropped down 4.62 μm in the absence of water vapor. The role of ethylene in the gas feed is to provide carbon source for the growth of CNT forest, however, the hydrocarbon simultaneously deactivates the catalytic particles via reduction. To lessen this disadvantageous effect, water vapor is added to the system, too, which is able to oxidize thus regenerate the catalyst particles continuously. Nevertheless, water vapor can react with the forming CNT forest reducing their height (CNT walls are very stable if well-graphitized so they can be more easily attacked from the ends) (Hernadi et al., 2001). Without water vapor not only the height of the CNTs was very low, but also the structural quality, probably because of the healing capacity of water vapor. It seems to be a reasonable explanation, nevertheless, in the literature of CCVD methods such interpretation occurs only in few cases (Sugime et al., 2018).

**Figure 3:**
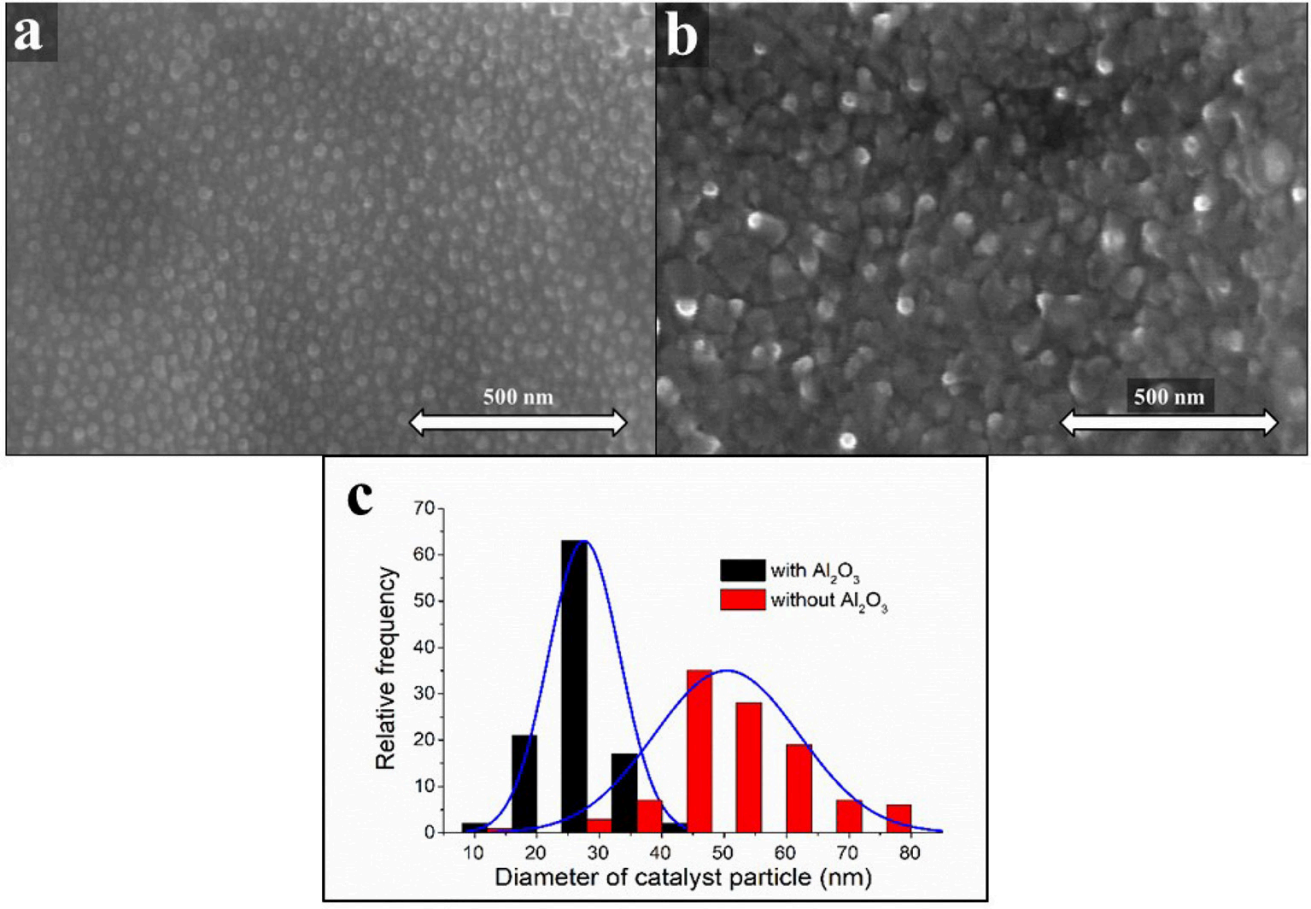
SEM images of CNT forests pre-synthesized with **(a)** and without Al_2_O_3_ oxide support **(b)**. Distribution of catalyst particle size with and without Al_2_O_3_ oxide support **(c)**.

The catalyst morphology, of a sample with a layer thickness of 5 nm, was investigated right after the heat treatment. During this blank synthesis, the carbon source was not allowed into the system. SEM images of the samples and a histogram of the particle size distribution can be seen in Figure 3. It can be concluded, that the supporting oxide layer has a significant influence on the distribution of catalyst particles on the surface of the substrate.

From the SEM images (Figure 3a), it can be observed, that the catalyst particles are separated when the oxide layer is deposited in advance onto the surface of the substrate and their average diameter is 27.5 ± 5.7 nm (Figure 3c). However, when there was no aluminum oxide layer on the substrate, the catalyst particles were aggregated probably as a result of the different wetting properties of the oxide and the metal (see Figure 3b) and their average diameter was 50.4 ± 11.6 nm (Figure 3c).

As in other metallic substrates, titanium can form special diphase type of alloys (α+β) with various metals and allows the formation of Ti_*x*_Me_*y*_ precipitates especially on the surface (Frommeyer, 2007). In this way, the essential condition of separated catalytic particles for seeding carbon nanotube growth at the very beginning of the CCVD reaction is ensured.

**Figure 4:**
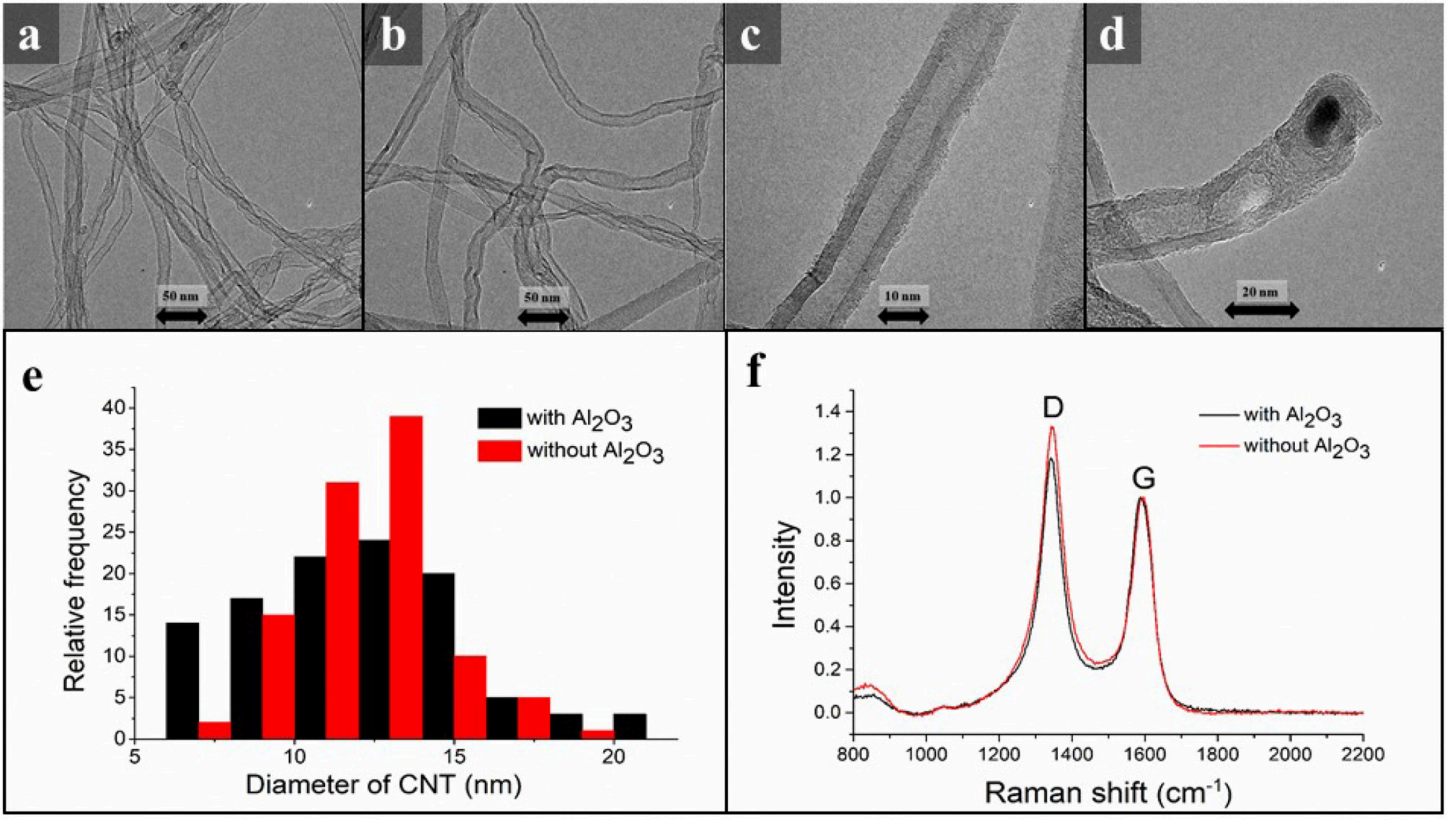
TEM images of carbon nanotubes synthesized with **(a)** and without Al_2_O_3_ oxide support **(b)**, with higher magnification **(c)**, of a catalyst particle at the end of a CNT **(d)**; the diameter distribution of carbon nanotubes grown in the presence or the absence of alumina layer **(e)**; Raman spectra of CNT forests synthesized with and without Al_2_O_3_ oxide support **(f)**.

Further structural analyses were performed on the CNT forests. TEM analysis was carried out to verify the quality of individual carbon nanotubes (Figures 4a,b). An observable difference was found between the graphitic properties of carbon nanotubes prepared with and without the oxide layer on the substrate. In accordance with Raman spectroscopy results, TEM investigations revealed that fewer defects can be detected in the CNT walls when an oxide layer is also deposited on the titanium substrate. HR-TEM images revealed that the CNTs were typically consisted of 8–9 walls in average (Figure 4c). The CNT with and increased number of walls showed much less graphitic features (Figure 4d). Furthermore, catalyst particles were predominantly not observed, which demonstrates that the carbon nanotube forest growth can be explained by the root mechanisms (Sugime et al., 2013; Yang et al., 2015). However, rarely particles were found at the end of the tube, (Figure 4d) not entirely excluding the tip growth mechanism. Analyzing the histogram it was concluded that the outer diameter of carbon nanotubes was between 12–13 nm for both samples (Figure 4e).

Raman Spectroscopy was used to determine the Raman shifts in the case of both samples. Based on the Raman spectra, only a small difference was observed, however, in the presence of Al_2_O_3_ the value of the I_*G*_/I_*D*_ peaks fraction was: I_*G*_/I_*D*_ = 1.18, while in the absence of Al_2_O_3_ it was I_*G*_/I_*D*_ = 1.33 (Figure 4f). According to literature data, samples with an oxide support exhibit higher-levels of graphitic properties.

### 3.2. The effect of catalyst ratio on the CNT forests

Secondly, the effect of the Fe:Co ratio of the catalyst was investigated regarding the CNT forest growth. As previously, samples were synthesized with and without the Al_2_O_3_ layer on the substrate, dividing the samples in two groups. Consequently, the catalyst ratios were changed as follows: Fe:Co = 1:3, 2:3, 1:1, 3:2, and 3:1.

The Al_2_O_3_ oxide layer proved to facilitate the growth of the CNT forests as it created a thin layer on the substrate, which suppressed diffusion and aggregation of the catalyst nanoparticles. It was found that the growth of CNT forests was dependent from the presence of the oxide layer, so we suppose that alumina may affect the deposition of the catalyst particles on the surface, as well as the formation of an Al-Fe alloy (Magrez et al., 2011).

**Figure 5:**
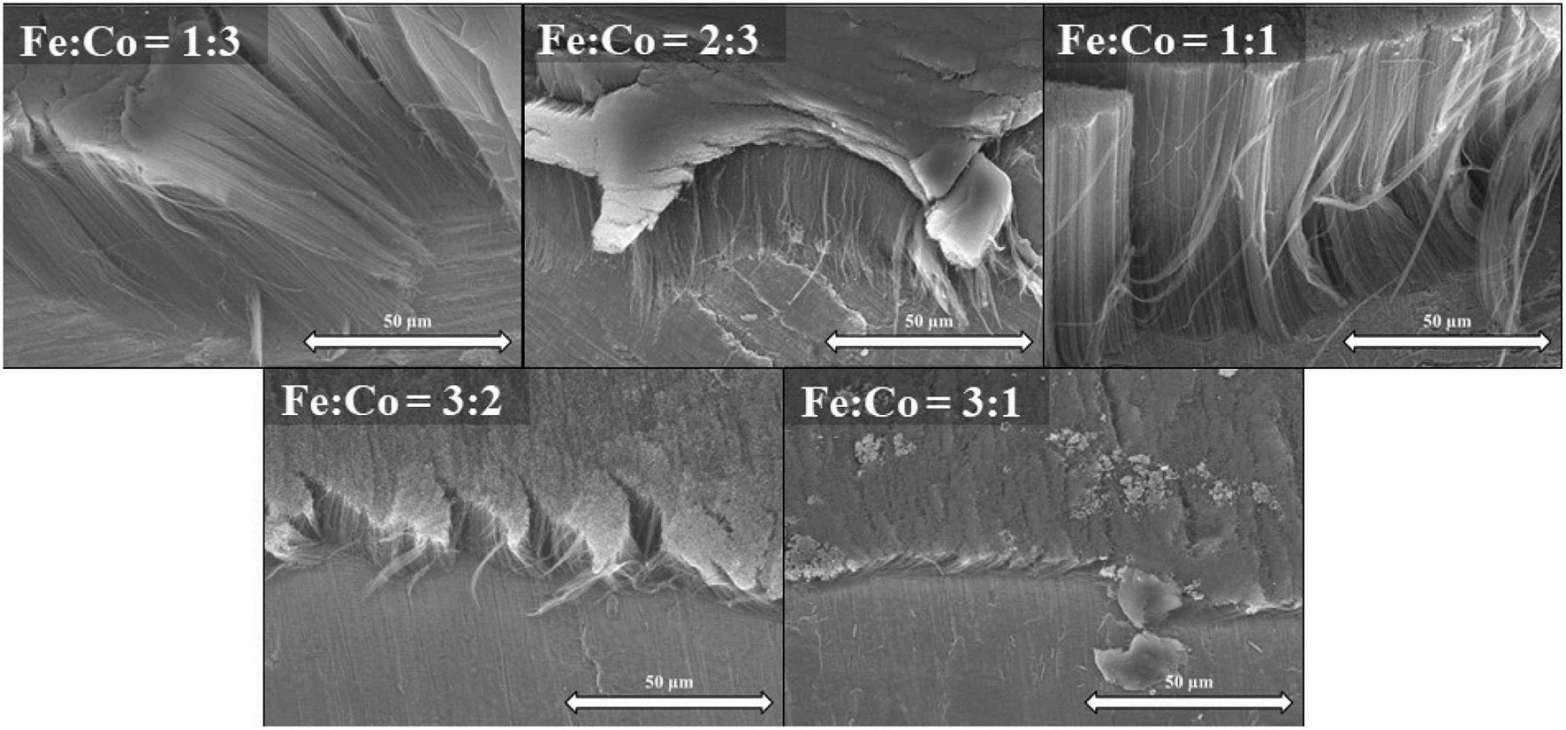
SEM images of CNT forests synthesized at various catalyst ratios with Al_2_O_3_ oxide layer on titanium support.

Based on literature data it is well known, that Ti has excellent wetting and thermal properties, along with being one of the best thermal interface materials. Therefore, it was feasible to deposit catalytic particles on its surface, ensuring the possibility to grow CNT forests directly on metal surfaces (Li et al., 2009). Accordingly, the effect of the Fe:Co ratio of the catalyst on the CNT forest growth was investigated *via* SEM (Figure 5).

From the SEM images (Figure 5), it was observed that the composition of the catalyst affects the heights of the carbon nanotube forests. However, at the same time, the orientation of the CNT forests was not changed significantly. The highest CNT forests 110 μm were attained with a 1:1 ratio. Surprisingly, this is in contrast with our former observations with an aluminum support (Szabó et al., 2017). It was believed that the height of the CNT forests grown with a 1:1 ratio is very similar to that of a 1:3 ratio. However, from the results in Figure 5 it is clear that with a 3:1 ratio, the minimum CNT forests height is obtained, namely 10 μm.

**Figure 6:**
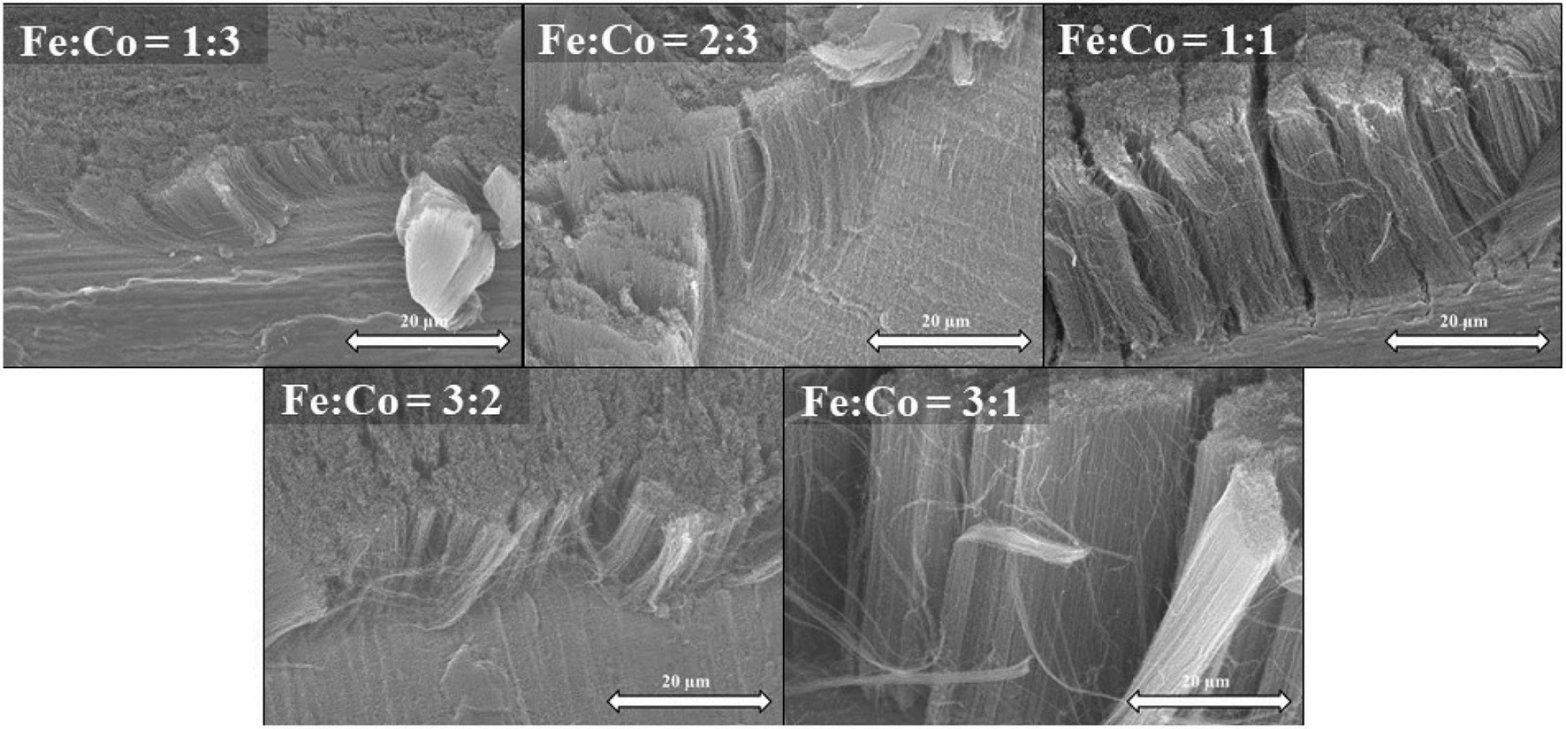
SEM images of CNT forests synthesized at various catalyst ratios without Al_2_O_3_ oxide layer on titanium support.

Same series of CNT growth, varying the catalyst ratio, were performed now employing titanium supports without the alumina layer. SEM images revealed (Figure 6), that different catalyst ratio have also affected the growth of carbon nanotube forests, but to a much less extent. While the height of the first four samples (1:3, 2:3, 1:1, and 3:2) was around 20 μm with small differences. The catalyst ratio Fe:Co = 3:1, enable the growth of a much higher CNT forest (62 μm). In contrast with former literature observations, the frequently used 1:1 ratio resulted in a much lower CNT forest, up to three times lower CNT forests than its 3:1 ratio counterpart.

**Figure 7:**
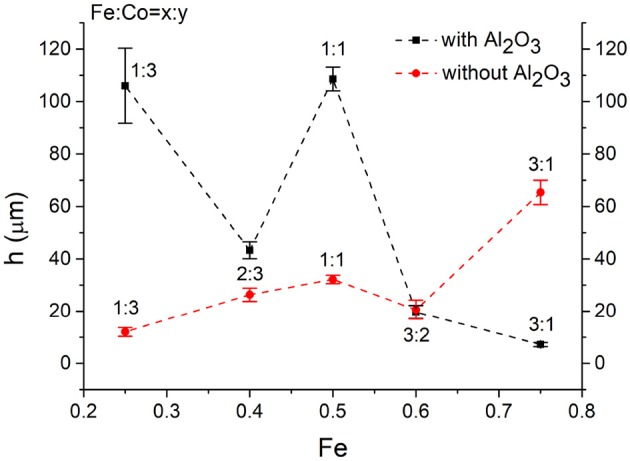
Heights of CNT forests with Al_2_O_3_ oxide support and without Al_2_O_3_ oxide support.

Summarizing the results from the two series, it was observed that in the presence of an insulating oxide layer on the surface of titanium substrate higher CNT forests were obtained. Interestingly, when the catalyst ratio was 2:3 or 3:2, the height of the CNT forests was relatively close in both cases, independently of the presence of the alumina layer. In the literature it can be found, that the oxide support has an advantageous effect on the substrate as the oxide layer can prevent the diffusion and aggregation of the catalyst nanoparticles, thus the dissolution of the reduced catalytic metal in the substrate can be prevented, as a result, on the oxide support separated nanoparticles on the substrate can act as catalyst. In our case when there was Al_2_O_3_ oxide support on the substrate, the highest CNT forest grew over the layers with 1:1 and 1:3 ratio as shown in the Figure 7. In the literature the most commonly used Fe:Co ratio is the 1:1 (Kaneko et al., 2012), however, in our system the 1:3 ratio produced similar height, probably due to the stronger interaction of cobalt oxide and alumina layer, keeping separated catalyst nanoparticles on the surface. In the second case when the substrate was applied without Al_2_O_3_ oxide layer, the maximum height of carbon nanotube forests was observed at the ratio of 3:1 as shown in the Figure 7 and practically the height increases linearly with iron content. Beside the above-mentioned Ti_*x*_Me_*y*_ precipitates, another occurrence might affect the growth mechanism of CNT growth, namely, increasing iron content in titanium substrate can enhance hydride formation (Dalebrook et al., 2013), which might promote carbon nanotube formation.

### 3.3. The effect of the oxide supports on performances of organic-inorganic halide perovskite photodetectors employing vertically aligned CNT forests as electrodes

Recently, CNT forests have been introduced as electrodes in sensitive hybrid perovskite photodetectors, able to detect visible light of nW intensities (Andričević et al., 2017). Therefore, light detector devices were fabricated using the aforementioned CNT forests and methylammonium lead tribromide (MAPbBr_3_) single crystals to study the effect of the presence of oxide layers on the detection properties. A heterojunction was fabricated by dry pressing a 5 mm MAPbBr_3_ single crystal on the top of CNT forests.

**Figure 8:**
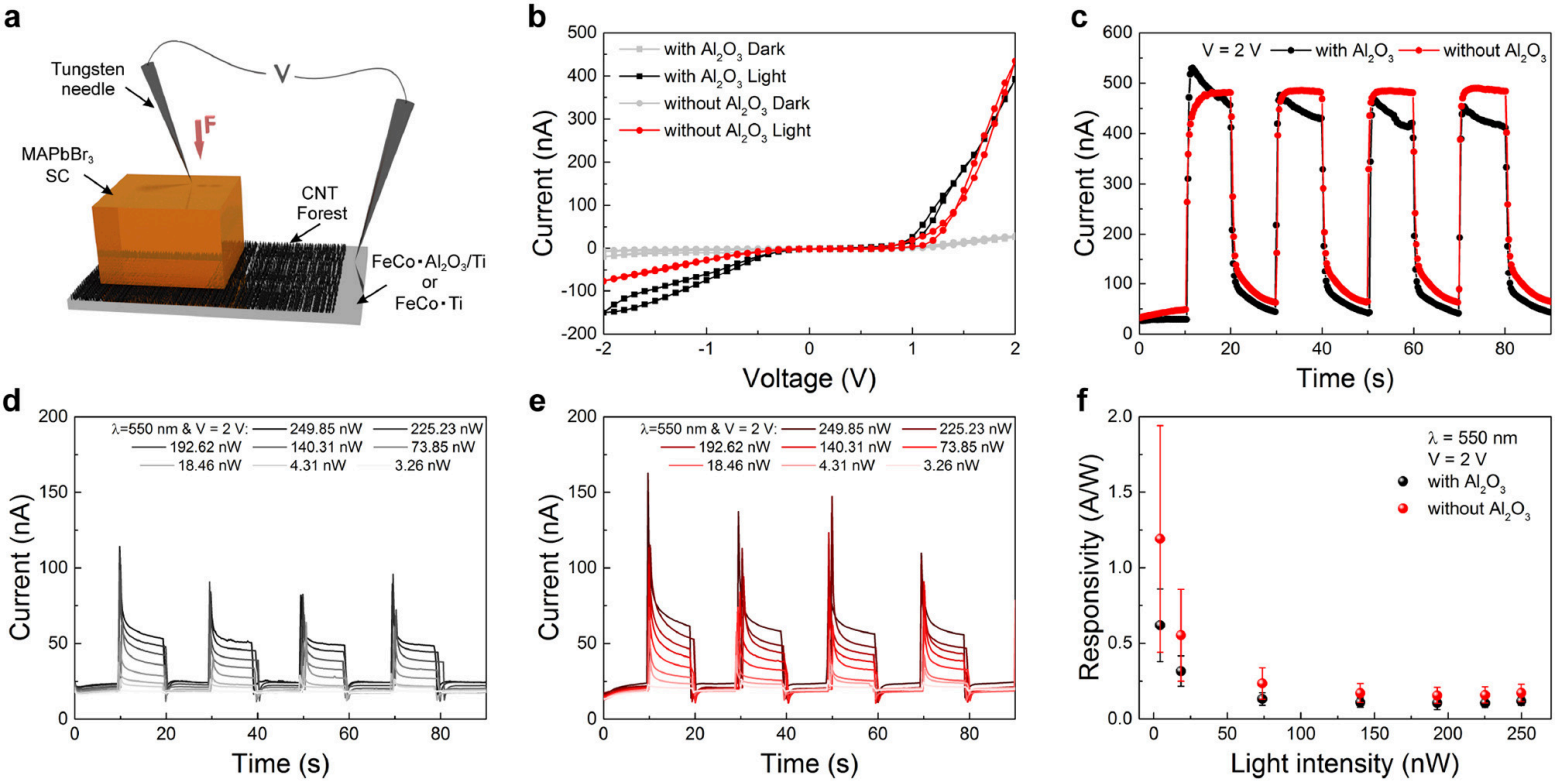
**(a)** Schematic illustration of the measurement setup. **(b)** Current-Voltage measurements in the dark and under visible light of intensity 1.02 mW/*cm*^2^. The voltage was swept from 0 to +2 V/-2 V and back. **(c)** Transient photoresponse on-off characteristics of the two devices under visible light illumination and a bias voltage of 2 V. **(d,e)** On-off characteristics of the photodetector with **(d)** and without **(e)** alumina layer under illumination with 550 nm light at different light intensities at a bias voltage of 2 V. **(f)** Responsivity of the photodetectors with (black) and without (red) alumina layer.

In order to measure the photodiode performance, tungsten needles, which served as electrical leads, were pressed on top of the surface of the perovskite single crystal and onto the Ti foil back electrode, respectively (Figure 8a). Current-Voltage (I–V) characteristics were measured for both samples in the dark and under white fluorescent light source with an intensity of 1.02 mW/cm^2^ (Figure 8b). Both devices exhibit diode-like characteristics. The presence of the aluminum oxide support does not differ the photocurrent of the forward bias region and the On-Off measurement significantly (Figure 8c). However, the photocurrent in reverse bias is smaller for the device without Al_2_O_3_, resulting in a higher diode ideality factor than its counterpart with the presence of an oxide layer. The photodetectors have been tested at low light intensities to determine the responsivity, a typical figure of merit for light sensing devices. On-Off measurements were performed under 550 nm green light for intensities ranging from 250 to 3 nW. The photocurrent increases with increasing light intensity for both devices with and without the oxide layer as seen in Figures 8d,e, respectively. Importantly, both devices detect light in the lowest intensity range of 3 nW, achieving reasonably high responsivities up to 1 A/W (Figure 8f).

## Conclusion

In conclusion, certain parameters during both catalyst preparation procedure and CCVD synthesis can strongly affect the growth of vertically aligned carbon nanotubes. Applying titanium plates as a substrate it was found that the presence of an alumina layer on the surface significantly modifies the morphology of the catalyst layer (before reaction), thus influencing the CNT forest growth. One could expect that titanium as a metallic substrate dissolves reduced Fe-Co nanoparticles during preliminary hydrogenation and in this way completely inhibits carbon deposition. However, in this study, titanium proved to show a different feature. Consequently, the formation of Ti_*x*_Fe-Co_*y*_ precipitates on the surface provided seeding for the CNT growth. Nevertheless, it was attested that the insulating layer plays a significant role in CNT forest formation: both the height and the quality of CNT forest depended on the initial structure of the catalyst layer. It was also pointed out that water vapor in the gas feed during CCVD considerably affects the same parameters of the final product. Structural characterization revealed important differences in CNT forests grown with or without alumina layer. However, these variances did not affect drastically the performance of photodetector devices employing these CNT forests as electrodes. These minor differences can be a result of many parameters such as graphitization of the CNTs, presence of alumina layer, density of CNT forest, fabrication conditions, etc. Therefore, we could postulate that the alumina layer does not play an important role as a current blocking layer. By eliminating the alumina deposition step, one might reduce the degree of complications, ultimately reducing the price of the optoelectronic device fabrication process.

## Author contributions

All the authors contributed to the discussion of the results and writing the manuscript. AS designed and performed the experiments, wrote the manuscript and contributed by taking the TEM images of CNT forests. PA and EH contributed by performing halide perovskite photodetectors of CNT forests. TG contributed by taking SEM images of CNT forests. KN contributed by Raman measurements of CNT forests. ZP contributed to the PLD technic of the catalyst layer. KH and LF conceived and designed the experiments.

### Conflict of interest statement

The authors declare that the research was conducted in the absence of any commercial or financial relationships that could be construed as a potential conflict of interest.
